# Cross-cultural adaptation of the Fresno Test for Turkish language

**DOI:** 10.1371/journal.pone.0245195

**Published:** 2021-01-08

**Authors:** Ozlem Serpil Cakmakkaya, Ayse Hilal Bati, Kerstin Kolodzie

**Affiliations:** 1 Department of Medical Education, Cerrahpasa Medical School, University of Istanbul-Cerrahpasa, Istanbul, Turkey; 2 Department of Medical Education, School of Medicine, Ege University, Izmir, Turkey; 3 Department of Anesthesia & Perioperative Care and Department of Epidemiology & Biostatistics, University of California San Francisco, San Francisco, CA, United States of America; Foundation IRCCS Neurological Institute C. Besta, ITALY

## Abstract

**Objective:**

National and international medical organizations and boards have recognized the importance of Evidence Based Medicine (EBM) and emphasized that EBM training should be included in medical education programs. Although some Turkish medical schools have developed and implemented EBM training programs, no validated Turkish language assessment tool has been available to compare the effectiveness of these training programs to national or international standards. The aim of this study is to cross-culturally adapt the Fresno Test, which is a validated English language tool utilized worldwide in the assessment of EBM training.

**Methods:**

This study is a cross-sectional validation study, which was performed in two stages: Cross-cultural adaptation of the Fresno Test into Turkish; and evaluation of the psychometric properties, validity, reliability and responsiveness, of the Turkish version of the Fresno Test.

**Results:**

The content validity of the test was evaluated by experienced physicians in the field of Evidence-Based Medicine, and the content validity index was 1.00. The Cronbach α coefficient was 0.78 on the post-test results. The intraclass correlation (ICC) coefficient and the kappa analysis were calculated to evaluate inter-rater reliability. The ICC coefficients ranged from 0.66 to 0.97 for pre- and post-test results. The Kappa coefficients were 1.00 for all pre-test and post-test questions except one post-test question which was 0.89.

The change score of the Fresno Test was used to evaluate responsiveness. The students' score of the Turkish Fresno Test was 49.9 ±18.2 pre-training and 118.9 ±26.3 post-training with a change of 69 points (95% CI, 63.9–74.2). The Cohen’s effect size was 3.04 (95% CI, 2.6–3.5) indicating a very large change in scores.

**Conclusions:**

The Turkish adapted Fresno Test used to evaluate students’ success and program effectiveness is a valid and reliable measurement tool. It will be of great benefit for the comparison of the effectiveness of Turkish education programs nationally and cross-culturally.

## Introduction

"Half of what you are taught as medical students will in 10 years have been shown to be wrong. And the trouble is none of your teachers knows which half."

Dr. Sidney Burwell, Dean at Harvard Medical School, 1956 [1]

### Medical knowledge changes at a very fast pace

Today we cannot tell our medical students how knowledge will change in the future, or in which direction. However, we can encourage students to be aware of the changes and teach them how to stay up to date during their careers. Teaching Evidence-Based Medicine (EBM), which aims to integrate reputable scientific research into clinical decision-making processes, promotes students' critical thinking skills and provides tools for a lifelong self-directed learning process [2]. National and international medical organizations and boards have recognized EBM’s importance and emphasized that EBM training should be included in medical education programs [3]. In Turkey, The Association of Medical Education Programs Evaluation and Accreditation includes acquisition and practice of EBM skills as an accreditation standard [4]. Although some Turkish medical schools have developed and implemented EBM training programs, no validated Turkish language assessment tool has been available to compare the effectiveness of these training programs to national or international standards. Turkish medical training institutions have instead relied on traditional, non-validated assessment methods, such as multiple-choice tests and/or written exams. Since our aim is to utilize evidence-based methods to develop effective EBM training, we sought to develop a more robust tool to measure students’ knowledge and skills acquisition, and to compare our EBM program at both the national and international level.

We conducted a search of all available EBM assessment tools. Although there are many tools in the English language, the number of validated tools is limited [5]. According to a systematic review, two EBM assessment tools, the Fresno Test and the Berlin Questionnaire, have strong psychometric properties, and they can be used in both formative and summative evaluations [5]. The Fresno Test is a validated English language tool utilized worldwide in the assessment of EBM education [5–7]. The original Fresno Test has content validity, good to excellent inter-rater reliability for all questions, and excellent internal consistency [8]. It includes open-ended questions, short answer questions and statistical calculation questions; the Berlin Questionnaire consists of multiple-choice questions [8,9]. More recently, another EBM tool was developed, the ACE (Assessing Competency in Evidence-Based Medicine) Tool [10]. This tool also has strong psychometric properties with high reliability and validity. However, it calls for a dichotomous response for each question. Of these three choices, we decided to adapt the Fresno Test. We believe that open-ended questions and statistical calculations can measure higher levels of cognitive thinking compared to multiple choice or dichotomous questions [11]. Additionally, according to the CREATE (Classification Rubric for Evidence-Based Practice Assessment Tools in Education) framework for classifying EBM assessment tools, the Fresno Test assesses more practical steps of EBM compared to other tools [6]. Therefore, we aimed to cross-culturally adapt the Fresno Test and to evaluate the psychometric properties of the adapted version with this study.

## Methods

Ethics committee approval was received from Cerrahpasa Medical School (Number: 83045809/604.01/02-264673). The developers of the original Fresno Test were contacted and permission for translation of the test was granted [8]. All volunteer students gave written informed consent before the pre-test and start of the course.

This cross-sectional validation study was performed in two stages: Cross-cultural adaptation of the Fresno Test and its grading rubric into Turkish; and evaluation of psychometric properties of the Turkish version of the Fresno Test for validation.

### Study population

Medical students from the 3rd, 4th and 5th year of Cerrahpasa Medical School were invited to participate in the newly developed EBM training program. We excluded the students in the first and second year because they did not have enough clinical training yet. Sixth year students were excluded as well because of their very busy schedule. An invitation was distributed by an online announcement on the students’ web site. Additionally, we informed the student representatives about the course and asked to share the information with their peers. Class size was limited to 25 students per year (75 in total) to allow for interactive learning.

### Fresno Test

The Fresno Test is a written test, used in undergraduate and postgraduate medical education for assessing medical students’ and health professionals’ knowledge and skills on EBM, rather than relying on self-report [8]. The recommended best use of Fresno Test is to measure change in knowledge and skills after instruction in EBM. It can assess the effectiveness of teaching in EBM and identify strengths and weaknesses of curricula and individuals. The test was initially evaluated in family practice residents and faculty members at the University of California San Francisco, Fresno Campus [8].

The Fresno Test consists of 12 questions, starting with two clinical scenarios. The first seven questions, which are about the clinical scenarios, require the student to formulate a focused research question, identify the most appropriate research design to answer the question, show knowledge of database searching, identify issues important for determining the relevance and validity of research articles, and discuss the magnitude and importance of research findings. The remaining five questions, (a series of statistical calculations and fill-in-the-blank questions) are independent of these scenarios. The maximum score that can be achieved is 212 [8]. The test takes approximately 30 minutes to complete.

A standardized grading rubric is used for scoring the answers [8]. The rubric contains explicit grading criteria for each question and describes levels of achievement in specific areas of performance such as creating a focused clinical question, understanding of different study designs or medical data base search. It is presented as a table, which is shaped like a matrix. The rows of the table represent four or five grading categories (not evident, minimal and/or limited, strong, excellent), each of which is associated with a point value for the first seven questions. Questions 8, 9, 10, 11 and 12 are short answer questions. Therefore, no different grading categories are needed for their scoring.

The content validity of the original Fresno Test was based on expert opinion [8]. Cronbach’s α level for internal reliability was reported to be 0.88. Inter-rater reliability was measured by a rating of two scorers for a single performance and ranged from 0.72 to 0.98 for individual items of the validation and development sets [8].

### Cross-cultural adaptation of the Fresno Test into Turkish

We followed well-established cross-cultural adaptation guidelines [12]. Additionally, we took into consideration literature reviews [13] and checklists on cross-cultural adaptation [14]. For the translation of the Fresno Test, we used a forward-translation back-translation design. Our expert group worked together during the whole adaptation process. This group consisted of three faculty members (Prof. of Anesthesiology; Prof. of Infectious Disease, Assoc. Prof of Anesthesiology and Medical Education PhD) with excellent command of English. All faculty members have experience in academic medicine, and all had several international publications in the leading journals of their area of expertise. Additionally, they are familiar to the English culture because of their international work experience. The head of the group is a clinical researcher who received training on clinical research and EBM, and who has international research experience.

We followed a stepwise systematic approach as presented in Table 1.

**Table 1:** The steps of cross-cultural adaptation of Fresno Test.

Steps	Details of the steps
1. Forward Translation	Expert group members translated the original Fresno Test and its rubric into Turkish independently.
2. Synthesis of translated versions and agreement on the first Turkish version	Based on their individual translations, all three versions of the test were consolidated, and the first agreed Turkish version was developed during a panel meeting.
3. Back Translation	A bilingual English translator translated the agreed Turkish version of the Fresno Test back to English.
4. Back translation review, agreement on the second Turkish version	The expert group of faculty members and bilingual English translator reviewed and compared the original and back-translated versions of the test. The group assessed whether the items translated correctly and checked the integrity of the meaning and the relevance of translated items. This group eventually reached consensus on the Turkish version of the Fresno test.
5. Pre-testing and focus group meetings	A pilot study was conducted to evaluate the meaning integrity and comprehensibility of the translated Fresno Test.
6. Expert group meeting; agreement on the third Turkish version	We checked whether the students perceived the meaning of each item in the same way as the original version, using a rephrasing approach.
7. Evaluation and the approval of the final version by a Turkish language expert	A Turkish language expert evaluated and approved the final version of the Turkish Fresno Test in terms of comprehensibility and meaning integrity.

### Pilot study (Step 5)

We created a focus group by inviting students from the 3rd, 4th and 5th year to be comparable with the target group of the study. Twenty-one students (7 students from each year, the mean age of the students was 21.9 ±1.03 years (Mean ±SD), female to male ratio was 7/14) volunteered to complete the test and to discuss the areas that they thought were problematic in terms of intelligibility and language. As recommended, we checked whether the students perceive the meaning of each item in the same way as the original version by using a rephrasing approach, and investigated the words or phrases where that failed to elicit an appropriate response [13]. Based on students’ feedback and suggestions for the items that were not well understood or misunderstood, wording was reviewed, and corrections were implemented. In this context, for question 5, a conjunction word was added for the strengthening of the meaning; for question 6 the tense of the question was changed, and for question 10 the English word “revealed” equivalent in Turkish was changed to another synonym.

Since the Fresno Test is a standardized, objective assessment tool of EBM knowledge rather than a self-assessment tool, we did not find important areas of adaptation related to the Turkish culture. We did change the English names to Turkish in the scenarios.

We did not remove any item or changed any item’s content. The test was finalized and administered to participants of the study for testing its psychometric properties.

### Testing the psychometric properties

Medical students who participated in a newly developed EBM training program completed the written Fresno Test. The test was given twice, once before EBM training and then after training completion. Students were allowed up to 40 minutes to complete the test. Open books were not allowed, and students were supervised during the exam.

#### EBM training program

We created a five-week EBM training program at Cerrahpasa Medical School to increase students' knowledge and skills in EBM. The program was named “School of Evidence-Based Medicine”. The program has been developed around the basic steps of EBM [15]. Lectures and workshop were given by three faculty members who were experts on EBM and biostatistics. Two of the faculty members were medical doctors and one was a professor of biostatistics with a PhD degree.

### Psychometric property validity

#### Face validity

Face validity assesses whether the test takers view the content of a test and its questions as relevant in the context in which the test being administered [16]. The expert group assessed the obviousness of items content, clarity of wording, layout and style of the test during a panel session.

#### Content validity

The content of the original Fresno Test is based on the domains of EBM [8]. The content validity of the Turkish version of the test was evaluated by a panel of five experienced physicians in the field of EBM. The Lawshe technique was used for the evaluation of content validity [17]. The Content Validity Ratio (CVR) of each item is calculated by using the following equation.

$$CVR=\frac{\mathrm{Ne}}{N/2}-1$$

**CVR:** Content Validity Ratio

**Ne:** Number of experts indicating items are essential

**N:** Total number of experts

The CVR ranges from -1 to +1. The Content Validity Index (CVI) of the test is calculated as the mean CVR for all items.

### Reliability

Evaluation of reliability of this study was comprised by assessment of internal consistency and inter-rater reliability.

#### Internal consistency

Internal consistency of the test was evaluated by calculating the pre- and post-test Cronbach-α reliability coefficients [18].

#### Inter-rater reliability

Inter-rater reliability is the degree to which two scorers rate a single performance similarly [8]. We randomly selected 20 students’ pre- and post-tests and two raters graded the same performances independently. Raters were blinded to students’ identities.

The intraclass correlation coefficient for continuous variables and the kappa analysis for categorical variables were calculated.

### Responsiveness

Responsiveness describes the sensitivity of an instrument to detect change over time [19]. We focused on the change score of the Fresno Test and calculated Cohen’s Effect Size, defined as the mean change score over the baseline standard deviation [20].

### Statistical analysis

#### Intra-class correlation (ICC)

A two-way mixed effect model was used for the calculation of ICC. In this model, while the raters are fixed, the tests are selected randomly from a certain test set. ICC less than 0.5 indicates poor reliability, 0.5–0.75 moderate reliability, 0.75–0.9 good reliability, and greater than 0.9 is excellent reliability [21].

#### Kappa analysis

We calculated kappa coefficients for categorical variables. The Kappa coefficient describes the agreement between raters. A Kappa coefficient between 0–0.20 indicates no agreement, 0.21 to 0.40 minimal, 0.41–0.59 weak, 0.60–0.79 moderate and 0.80–0.90 strong, above 0.90 is almost perfect agreement [22].

Descriptive statistics were used to present demographic data.

The paired t-test was used to compare pre-test and post-test scores. In the statistical analysis, an α error was accepted at a level of 0.05.

For statistical analysis STATA SE 14.2 (StataCorp LP, College Station, TX) and SPSS for Windows, Version 23.0. (SPSS Inc., Chicago, IL) were utilized.

De-identified underlying data can be found as S1 and S2 Tables.

## Results

A total of 76 students from the 3^rd^, 4^th^ and 5^th^ year of medical school voluntarily consented and were enrolled in the School of Evidence-Based Medicine. Of those, one student did not attend the whole program including pre-test taking and three students did not complete the post-test. These four students were excluded from the analysis. Of the remaining 72 students, 29 were from the 3^rd^, 19 from the 4^th^ and 24 from the 5^th^ year of medical school. The mean age of the students was 21.6 ±1.05 years (Mean ±SD). Female to male ratio was 39/33 (54.2% female).

### Psychometric properties of the Turkish version of the Fresno Test

#### Validity results. Face validity

A standard expert-panel process confirmed the face validity of all items of the Turkish version of the Fresno Test. Experts agreed that the items of the test are appropriate, and relevant to EBM. All of them confirmed that the clarity of wording, layout and style of the test are adequate.

#### Content validity

Content validity was assessed based on the expert opinions of the five panel members. The CVR was calculated to be 1.0 for each item. Therefore, the mean CVR across all items (CVI) was 1.0 as well.

### Reliability results

#### Internal consistency

The Cronbach-α coefficient was calculated to be 68.2 for the pretest and 78.4 for the post-test.

#### Inter-rater reliability

ICCs ranged between 0.66 and 0.95 for the pre-test and 0.83 and 0.97 for the post-test. The highest ICC was 0.97 for the study design question in the post-test. The lowest ICC was 0.66 for the seventh question of the pre-test, which examined the effect size; for the same question, the post-test coefficient was 0.87. The ICCs show that inter-rater reliability is overall good to excellent for continuous variables (Table 2: Question 1–7).

**Table 2:** Psychometric measurements of questions 1–7.

No	Assessment Area	Inter-rater Reliability Intraclass Correlation Coefficients (ICC) *		Content Validity Ratio (CVR)
		Pre-test (n = 72)	Post-test (n = 72)	
1	Writing a focused clinical question (PICO)	0.92 (0.81–0.97)	0.97 (0.92–0.99)	1.00
2	Information sources	0.86 (0.69–0.94)	0.94 (0.85–0.98)	1.00
3	Study designs	0.95 (0.88–0.98)	0.87 (0.71–0.95)	1.00
4	Search strategy	0.93 (0.82–0.97)	0.91 (0.78–0.96)	1.00
5	Relevance	0.78 (0.53–0.91)	0.83 (0.61–0.93)	1.00
6	Validity	0.83 (0.61–0.93)	0.88 (0.71–0.95)	1.00
7	Magnitude of effect	0.66 (0.32–0.85)	0.87 (0.71–0.95)	1.00

* Intraclass Correlation Coefficient (CI 95%).

The Kappa coefficients were 1.0 for all pre- and post-questions, except for the Confidence Interval topic (Question 10) in the post-test, which was 0.89. Kappa coefficients indicated excellent consistency ((Table 3: Question 8–12).

**Table 3:** Psychometric measurements of questions 8–12.

No	Assessment Area	Inter-rater Reliability Kappa Coefficients *		Content Validity Ratio (CVR)
		Pre-test (n = 72)	Post-test (n = 72)	
8	a) Sensitivity	1.00 ±0.22	1.00 ±0.22	1.00
	b) Specificity	1.00 ±0.22	1.00 ±0.22	1.00
	c) Positive Predictive Value	1.00 ±0.22	1.00 ±0.22	1.00
	d) Negative Predictive Value	1.00 ±0.22	1.00 ±0.22	1.00
	e) Positive Likelihood Ratio	1.00 ±0.22	1.00 ±0.22	1.00
9	a) Absolute Risk Reduction	1.00 ±0.22	1.00 ±0.22	1.00
	b) Relative Risk Reduction	1.00 ±0.22	1.00 ±0.22	1.00
	c) Number Needed to Treat (NNT)	1.00 ±0.22	1.00 ±0.22	1.00
10	Confidence Interval	1.00 ±0.22	0.89 ±0.22	1.00
11	Identification of Best Study Design for Diagnosis	1.00 ±0.22	1.00 ±0.22	1.00
12	Identification of Best Study Design for Prognosis	1.00 ±0.22	1.00 ±0.22	1.00

* Kappa coefficient ±SD.

### Responsiveness

The students had a score of 49.9 ± 18.2 in the adapted Fresno EBM test prior to participating in the School of Evidence-Based Medicine. The average post training score was 118.9 ±26.2, an increase by 69 ±21.9 points (95% CI: 63.9–74.2).

Cohen’s effect size was 3.06 (95% CI: 2.6–3.5) for responsiveness. This is considered a very large change [23].

## Discussion

Our study shows the feasibility to cross-culturally adapt the Fresno Test to the Turkish language. The Turkish version of the Fresno Test exhibits very good psychometric properties to measure students’ EBM knowledge and skills.

The Fresno Test is one of the most used EBM tests across the world [5,7]. The original test has content validity, good to excellent inter-rater reliability for all questions, and excellent internal consistency [5,8]. The psychometric properties of the Turkish version are consistent with the original and previously published cross-culturally adapted tests [8,24,25].

The Fresno Test has previously been cross-culturally adapted to Spanish [25], Dutch [26], Chinese [27], and Brazilian-Portuguese languages [28], and validity-reliability studies were performed. Study populations were usually physicians or other health care professionals [25–28]. Some studies modified the Fresno Test in order to be more suitable for their study populations [26,28]. All adapted tests showed good psychometric properties that were comparable to our results and to the original test [8]. The mutual motivation of these studies—including ours—was the lack of a validated EBM tool in their own languages. Validated EBM tools are needed for assessing the effectiveness of EBM programs and curricula; assessing the trainees’ competencies in EBM; the comparison of national curricula with other countries’ curricula; and comparison of different teaching methods. The Fresno Test best fit our needs to assess EBM training at Cerrahpasa Medical School. The new adaptation of the Fresno Test will now allow not only for national but also international comparisons of students’ performance in EBM.

During the adaptation study we followed the recommendations for the cross-cultural adaptation processes but there is no clear consensus on the superiority of one method over another [13]. Additionally, these recommendations are commonly developed for the adaptation of health status self-administered questionnaires [14]. The Fresno Test is a standardized, objective knowledge assessment tool rather than a self-assessment questionnaire of self-reported outcomes. These characteristics might make the adaptation process easier and more sound. The relevance and comprehensiveness of the Turkish Fresno Test, which are the main components of the content validity, are similar with the original test. Also, our reliability results, internal consistency, and inter-rater reliability are comparable to the original test.

During the study period, we faced some challenges using the Fresno Test that might limit its implementation for large-group teaching activities. Grading of the Fresno Test requires more time compared with multiple-choice tests [5]. In larger student groups this must be taken into consideration. An analytical scoring tool, the Grading Rubric, is used to evaluate the test’s open-ended responses. The Rubric ensures objective evaluation, but it is not designed to maximize ease of use for the teachers or test raters. Raters are required to have experience in the field of EBM. For example, a question that tests the understanding of confidence intervals relies on the rater’s knowledge of this topic. Without rater’s expertise, interpretation of the answers might be difficult and inconsistent. Raters also need to be trained on the use of the grading rubric as emphasized in previous similar studies to achieve good inter-rater reliability [25,29]. Even though we included a training session for the rates before grading, a training deficit might be the reason for the only moderate pretest ICC for the questionnaire item “magnitude of effect”.

Because the School of Evidence-based Medicine was the first standardized EBM training program at our institution, the students’ EBM knowledge and skills were similar although they were from different medical school years. Therefore, we did not have novice and expert participants and we could not assess a ceiling effect.

## Conclusions

Our study indicates that our adaptation of the Fresno Test to Turkish might become a useful tool for the evaluation of the effectiveness of evidence-based medicine programs in Turkey. It will be of great benefit for the comparison of the effectiveness of the education programs nationally and cross-culturally.

## Supporting information

S1 Table Turkish Fresno Test data. 72 Students, deidentified.
(XLSX)

S2 Table Interrater reliability data. 20 Students, deidentified.
(XLSX)

S1 File (PDF)

S2 File (PDF)
