# Correction: Cross-cultural adaptation of the Fresno Test for Turkish language

**DOI:** 10.1371/journal.pone.0250835

**Published:** 2021-04-22

**Authors:** Ozlem Serpil Cakmakkaya, Ayse Hilal Bati, Kerstin Kolodzie

There is an error in affiliation 1 for author Ozlem Serpil Cakmakkaya. The correct affiliation 1 is: Department of Medical Education, Cerrahpaşa Medical Faculty, Istanbul University-Cerrahpaşa, Istanbul, Turkey.
