# Supplementary material for: Cross-cultural adaptation of the Fresno Test for Turkish language
Source: PLoS One. 2021 Jan 8;16(1):e0245195. doi: 10.1371/journal.pone.0245195 (PMC7793267; doi:10.1371/journal.pone.0245195)
Supplement: S1 File — (PDF) [file pone.0245195.s003.pdf]

KANITA DAYALI TIP FRESNO TESTİ

TEST YÖNERGESİ

Kanıta Dayalı Tıp (KDT) uygulaması, belli ölçüde temel bilgiyi ve tıbbi literatürü araştırma ve değerlendirme ile ilgili becerileri kapsar. Kanıta Dayalı Tıp becerilerinizin düzeyini değerlendirmek için bir değerlendirme testi geliştirilmiştir. Bu test yedisi kısa cevaplı, ikisi bir dizi matematik hesaplama gerektiren ve üçü de boşluk doldurma şeklinde olan toplam 12 soru içermektedir. Lütfen testi 30 dakikalık tek bir oturumda tamamlayınız.

1-4. soruları aşağıdaki klinik senaryolara göre yanıtlayınız:

- Yakın zamanda doğum yapıp sağlıklı bir bebeği olan Aysel Hanım'ı gördünüz. Aysel Hanım bebeğini emzirmeyi planlıyor fakat aynı zamanda doğum kontrolü için oral kontrasepsif başlamak istiyor. Genel olarak kombine oral kontraseptif (östrojen+progesteron) reçete etmeyi tercih ediyorsunuz fakat bunun anne sütü yapımını sadece progesteron içeren haplara göre daha olumsuz etkileyebileceğini duymuştunuz.

- Ali, primer enürezisi olan 11 yaşında bir çocuktur. Bu problemin getirdiği rahatsızlık ve mahcubiyetten artık çok sıkılmıştır. Olası nedenler olarak üriner yol anomali ve enfeksiyonlarını elediniz. Yatak ıslatma alarmı önermeyi düşünüyorsunuz ancak bir meslektaşınız bunların yararsız olduğunu düşündüğünü söylüyor ve İmipramin veya Desmopressin ile tedavi etmenizi öneriyor.

1. Her iki hasta görüşmesi ile ilgili uygun yanıtı bulmak için literatür taramanıza yardımcı olacak birer klinik soru yazınız ve literatürde bulduğunuz makaleler arasından en iyi makaleyi seçiniz.

2. Klinisyenler bu tür soruların cevabını bulmak için nereye başvurabilirler? Mümkün olduğunca çok sayıda bilgi kaynağının ismini verin. Klinik uygulamada başvurulan genel bilgi kaynaklarından bazılarının diğerlerinden daha iyi olduğunu düşünebilirsiniz ancak bu kaynakların güçlü ve zayıf yanları konusundaki farkındalığınızı göstermek için mümkün olduğunca açıklama yapın. İsmini verdiğiniz her bir bilgi kaynağı için en önemli avantaj ve dezavantajları açıklayınız.

3. Yukarıdaki klinik senaryolardan bir tanesine odaklanın (emzirme ve oral kontraseptifler ya da yatak ıslatma alarmı). Sizce hangi tür bir çalışma (çalışma tasarımı-study design) bu soruyu en iyi şekilde yanıtlar? Neden?

4. Bu sorulardan birisi ile ilgili özgün bir araştırmayı Medline'da arayacak olsanız; nasıl bir tarama stratejisi izlerdiniz, açıklayınız. Hangi konular ve araştırma kategorilerini (alanları) tarayacağınızı belirtiniz. Neden bu yaklaşımı seçtiğinizi açıklayınız. Taramanızı sınırlamanız (limit) gerekirse bunu nasıl yaparsınız, açıklayınız ve gerekçesini anlatınız.

5. Bu sorularla ilgili özgün bir araştırma makalesi bulduğunuzda, bunun araştırdığınız konu ile ilgili olup olmadığını (relevant) saptamak için araştırmanın hangi özelliklerine dikkat edersiniz? Örnekler veriniz. (6 ve 7. sorularda çalışmanın geçerli bir çalışma olup olmadığını ve bulguların ne kadar önemli olduğu sorulacaktır. Bu soru için sizin pratik uygulamanızla gerçekten ilgili olup olmadığını nasıl belirleyeceğinizi düşününüz).

6. Yaptığınız taramada sorularınızla ilgili özgün bir araştırma makalesi bulduğunuzda, araştırmanın hangi özellikleri bulgularının geçerli olduğunu düşünmenizi sağlar? Örnekler veriniz (İlgili olma konusunu zaten ele aldınız, 7. soruda ise bulguların önemini nasıl değerlendireceğiniz sorulacaktır. Bu soru için çalışmanın geçerliğine odaklanınız).

7. Sorularınızla ilgili özgün bir araştırma makalesi bulduğunuzda, bulguların hangi özellikleri onların kuvvet (etki büyüklüğü) ve istatistiksel anlamlılığını belirlemenizi sağlar? Örnekler veriniz (İlgili olma ve geçerlilik konusunu zaten ele aldınız. Bu soru için çalışmada bildirilen bir etkinin büyüklüğünü ve

anlamını nasıl belirleyeceğinize odaklanınız).

8. Pulmoner emboli tanısı koymada arter kan gazı değerlerinin tanısal duyarlılığı ile ilgili yeni yapılan bir çalışmaya, pulmoner emboli kuşkusu olan 212 hasta dahil edilmiştir, daha sonra bu hastaların 49'unda pulmoner emboli olduğu saptanmıştır. Pulmoner embolisi olanların 41'inde anormal alveol-arter oksijen gradiyenti ((A-a) DO_2) olduğu görülmüştür. Pulmoner embolisi olmadığı saptanan 163 hastanın 118'inde anormal (A-a) DO_2 olduğu belirlenmiştir.

   Bu sonuçlara dayanarak pulmoner emboli için aşağıdaki hesaplamaları yapınız (Her biri 4 puan).

   - (A-a) DO_2 duyarlılığı: (sensitivity):
   - (A-a) DO_2 özgüllüğü (specificity):
   - (A-a) DO_2 pozitif öngörü değeri (positive predictive value):
   - (A-a) DO_2 negatif öngörü değeri (negative predictive value):
   - Anormal (A-a) DO_2 için pozitif olabilirlik oranı (positive likelihood ratio):

9. Yakın zamanda yapılan randomize bir çalışmada koroner arter hastalığı olan diyabet hastaları 5 yıl takip edilmiştir. Pravastatin ile tedavi edilen hastaların % 29'u ile plasebo grubundakilerin %37'sinin tekrar eden koroner hadiseden yakındığı saptanmıştır.

   Tekrarlayan hadise için aşağıdaki değerleri belirleyiniz (Her biri 4 puan)

- Mutlak risk azalması (absolute risk reduction):
- Rölatif risk azalması (relative risk reduction):
- Bir tekrarlayan hadiseyi önlemek için tedavi edilmesi gereken hasta sayısı (number needed to treat: NNT):

10. Yakın zamanda yapılan HERS çalışmasında östrojen desteği alan kadınlar ile plasebo alan kadınlar karşılaştırılmıştır. Sonuçlar, östrojen kullanan kadınlarda venöz tromboembolik hadise için rölatif riskin 2.89 olduğunu göstermiştir. Bu sonuç, östrojen tedavisinin koroner arter hastalıkları için risk oluşturduğunu düşündürmektedir. Bu iki tedavi grubu arasındaki farklılığın istatistiksel olarak anlamlı olup olmadığını belirlemek için güven aralığına bakıyoruz. Venöz trombolembolik hadise oranının gerçekten (istatistiksel olarak) bu iki tedavi grubu için farklı olduğu çıkarımını destekleyecek güven aralığına bir örnek veriniz. (4 puan)

11. Size göre tanıya (diagnosis) yönelik bir araştırma için en iyi çalışma tasarımı nedir? (4 puan)

12. Size göre prognoza (prognosis) yönelik bir araştırma için en iyi çalışma tasarımı nedir? (4 puan)
