# Supplementary material for: Cross-cultural adaptation of the Fresno Test for Turkish language
Source: PLoS One. 2021 Jan 8;16(1):e0245195. doi: 10.1371/journal.pone.0245195 (PMC7793267; doi:10.1371/journal.pone.0245195)
Supplement: S2 File — (PDF) [file pone.0245195.s004.pdf]

Fresno Kanıta Dayalı Tıp Testi

Puanlama Anahtarı

Kanıta Dayalı Tıp (KDT) uygulaması, belli ölçüde temel bilgiyi ve tıbbi literatürü araştırma ve değerlendirme ile ilgili becerileri kapsar. Kanıta Dayalı Tıp becerilerinizin düzeyini değerlendirmek için bir değerlendirme testi geliştirilmiştir. Bu test yedisi kısa cevaplı, ikisi bir dizi matematik hesaplama gerektiren ve üçü de boşluk doldurma şeklinde olan toplam 12 soru içermektedir. Lütfen testi 30 dakikalık tek bir oturumda tamamlayınız.

1-4. soruları aşağıdaki klinik senaryolara göre yanıtlayınız:

Yakın zamanda doğum yapıp sağlıklı bir bebeği olan Aysel Hanım'ı gördünüz. Aysel Hanım bebeğini emzirmeyi planlıyor fakat aynı zamanda doğum kontrolü için oral kontrasepsif başlamak istiyor. Genel olarak kombine oral kontraseptif (östrojen+progesteron) reçete etmeyi tercih ediyorsunuz fakat bunun anne sütü yapımını sadece progesteron içeren haplara göre daha olumsuz etkileyebileceğini duymuştunuz.

Ali, primer enürezisi olan 11 yaşında bir çocuktur. Bu problemin getirdiği rahatsızlık ve mahcubiyetten artık çok sıkılmıştır. Olası nedenler olarak üriner yol anomali ve enfeksiyonlarını elediniz. Yatak ıslatma alarmı önermeyi düşünüyorsunuz ancak bir meslektaşınız bunların yararsız olduğunu düşündüğünü söylüyor ve İmipramin veya Desmopressin ile tedavi etmenizi öneriyor.

1. Her iki hasta görüşmesi ile ilgili uygun yanıtı bulmak için literatür taramanıza yardımcı olacak birer klinik soru yazınız ve literatürde bulduğunuz makaleler arasından en iyi makaleyi seçiniz.

Emzirme/kontrasepsiyon sorusu için puanlama listesi. (Şüpheye düşerseniz yazılanın spesifik klinik literatür taramanıza katkıda bulunup bulunmayacağını göz önünde bulundurun.)

	**Popülasyon**	**Müdahale**	**Karşılaştırma**	**Sonuç**
Mükemmel (3 puan)	İlgili çeşitli tanımlayıcılar	İlgilenilen spesifik müdahaleyi içerir;	Hasta oral kontraseptif kullanmak istediği için bununla ilgili spesifik bir alternatifi tanımlar	Hasta için objektif ve anlamlı sonuç
	Örneğin: “doğum sonrası kadın”, “emzirme/emziren anne” veya “doğum kontrolü isteyen emziren anne” veya anne sütü ile beslenen yenidoğan”<br>Not: “emziren kadın” iki tanımlayıcı olarak kabul edilir.	Örneğin; Kombine kontraseptifler (östrojen ve progesteron) veya “östrojen” gibi kontrasepsiyonun spesifik bileşenleri	Örneğin; sadece progesteron içeren doğum kontrolü	Örneğin; “bebek büyüme hız”ı, “emzirme sayısı”, “bırakma” veya “bebek tokluğu ile ilgili anne memnuniyeti” veya “süt akışı
Güçlü (2 puan)	Yukarıdaki örneklerdeki gibi bir tane uygun tanımlayıcı	Doğum kontrolü veya müdahalenin türünden	Spesifik bir karşılaştırma grubundan bahseder	Spesifik olmayan sonuç

		bahseder,		
	Örneğin, “kadın” veya “bebek” veya “emzirme”	Örneğin, oral kontraseptifler veya hormonlar	Örneğin; plasebo veya doğum kontrolünün spesifik bir türü veya oral kontraseftif kullanmayan anneler	Örneğin; “süt” veya “emzirme” veya “süt miktarı” gibi klinik anlamlılık belirtmeyen hasta odaklı sonuç Örneğin “süt miktarı” veya “sütün kimyasal bileşimi” veya “anne sütü yapımı”
Kısıtlı (1 puan)	Muhtemelen aramaya katkısı olmayacak genel bir tanımlayıcı	Muhtemelen aramaya katkısı olmayacak müdahaleden bahseder	Muhtemelen aramaya katkısı olmayacak karşılaştırmadan bahseder.	Sonuçtan bahseder ancak çok geneldir ve muhtemelen aramaya katkısı olmayacaktır
	Örneğin; “hasta”	Örneğin; “yöntemler” , “seçenekler”, “tedaviler”	Örneğin; “Diğer yöntemlerle karşılaştırıldığında” (Not: Müdahale sütununda “çeşitli tedavi seçenekleri” gibi spesifik olmayan çoğul bir terim	Örneğin; “etkiler”, “sonucu değişmesi”

			kullanıldığında sadece bir kez sayılmalıdır)	
Belirsiz (0 puan)	Yukarıdakilerin hiçbiri mevcut değil	Yukarıdakilerin hiçbiri mevcut değil	Yukarıdakilerin hiçbiri mevcut değil	Yukarıdakilerin hiçbiri mevcut değil

Yatak ıslatma sorusu için puanlama listesi. (Şüpheye düşerseniz yazılanın spesifik klinik literatür taramanıza katkıda bulunup bulunmayacağını göz önünde bulundurun.)

	**Popülasyon**	**Müdahale**	**Karşılaştırma**	**Sonuç**
Mükemmel (3 puan)	İlgili çeşitli tanımlayıcılar<br><br>Örneğin; "primer enürezisi olan erkek çocuk", spesifik yaş grubu, cinsiyet, enfeksiyon ve anatomik anomalilerin elenmesi<br>Not: "primer enürezis" iki tanımlayıcı olarak kabul edilir.	İlgilenilen spesifik müdahaleyi içerir;<br><br>Örneğin; yatak ıslatma alarmı	Spesifik bir alternatif tedavi tanımlar<br><br>Örneğin; "desmopresin asetat" veya "imipramin" veya "anti-depresanlar"	Hasta için objektif ve anlamlı bir sonuç<br><br>Örneğin; altını ıslatmadığı gece sayısı
Güçlü (2 puan)	Yukarıdaki örneklerdeki gibi bir tane uygun tanımlayıcı	Ayrıntılarını vermeden müdahalenin türünden bahseder	Spesifik bir karşılaştırma grubundan bahseder	Klinikle ilgili bir ölçü belirtilmeden hasta odaklı sonuç

	Örneğin; “enüresis” veya “çocuk”	Örneğin; “Davranış biçimlendirme”	Örneğin; “plasebo” veya “tıbbi tedavi” veya “tedavi uygulamamak”	Örneğin; “idrar çıkışı”
Kısıtlı (1 puan)	Muhtemelen aramaya katkısı olmayacak genel bir tanımlayıcı<br><br>Örneğin; “hasta”	Muhtemelen aramaya katkısı olmayacak müdahaleden bahseder<br><br>Örneğin; “yöntemler” , “seçenekler”, “tedaviler”	Muhtemelen aramaya katkısı olmayacak karşılaştırmadan bahseder<br><br>Örneğin; “Diğer yöntemlerle karşılaştırma”<br><br>(Not: Müdahale sütununda “çeşitli tedavi seçenekleri” gibi spesifik olmayan çoğul bir terim kullanıldığında sadece bir kez sayılmalıdır)	Sonuçtan bahseder ancak çok geneldir ve muhtemelen aramaya katkısı olmayacaktır<br><br>Örneğin; “etkin”, “iyileştirme”, “başarı”, “sonuçta değişiklik”
Belirsiz (0 puan)	Yukarıdakilerin hiçbiri mevcut değil	Yukarıdakilerin hiçbiri mevcut değil	Yukarıdakilerin hiçbiri mevcut değil	Yukarıdakilerin hiçbiri mevcut değil

2. Klinisyenler bu tür soruların cevabını bulmak için nereye başvurabilirler? Mümkün olduğunca çok sayıda bilgi kaynağının ismini verin. Klinik uygulamada başvurulan genel bilgi kaynaklarından bazılarının diğerlerinden daha iyi olduğunu düşünebilirsiniz ancak bu kaynakların güçlü ve zayıf yanları konusundaki farkındalığınızı göstermek için mümkün olduğunca açıklama yapın. İsmini verdiğiniz her bir bilgi kaynağı için en önemli avantaj ve dezavantajları açıklayınız.

	**Kaynak çeşitliliği**	**Kolaylık**	**Klinik İlgililik**	**Geçerlik**
Mükemmel (6 puan)	En az dört kaynak sıralamıştır.<br>Tipleri şunlardır:	Tartışma kolaylıkla ilgili en az iki spesifik konuyu içerir veya farklı iki kaynağı tartışırken aynı konudan bahseder.<br>Konular şunları içerebilir:	Tartışma klinik alaka ile ilgili en az iki spesifik konuyu içerir veya farklı iki kaynağı tartışırken aynı konudan bahseder.<br>Konular şunları içerebilir:	Tartışma geçerlik ile ilgili en az iki spesifik konuyu içerir veya farklı iki kaynağı tartışırken aynı konudan bahseder.<br>Konular şunları içerebilir:
	• Orijinal literatür içeren elektronik veri tabanları (Medline, Embase, CINAHL)<br>• Dergiler (JAMA, NEJM)<br>• Kaynak kitap (textbook) (Merck, Harrisons,	• Maliyet (“bedava”, “sadece abonelikle”)<br>• Hız (“hızlı”, “zaman alıcı”)<br>• Tarama kolaylığı (“taramanın nasıl daraltacağını bilinmeli,” “gezinme kolaylığı”)	• Klinikle alakalı sonuçlar<br>• Klinik uygulama için yazılanlar (örneğin; “ilgili”, “yan etkiler hakkında bilgi” veya “hasta bilgilendirme sayfaları mevcut”)<br>• İhtisasa uygun odaklanma (“aile hekimlerine yönelik”)	• Geçerliğin kesinliği (örneğin; “kalite belirli değil” veya “araştırılmamış” veya “eleştirel olarak değerlendirilmesi gerekir”)<br>• Kanıta dayalı yaklaşım (örneğin: “Kanıta dayalı” veya

	monographs)<br>• Sistematik derlemeler (Cochrane)<br>• Kanıta Dayalı Tıp yayınları veya önceden değerlendirilmiş bilgilerin bulunduğu veri tabanları (Best Evidence, InfoRetriever, DynaMed, EBM, ACPJC, EBP, Clinical Evidence)<br>• Tıbbi web siteleri (MDConsult, PraxisMD, SumSearch)<br>• Genel internet taraması (google, yahoo)<br>• Klinik kılavuzlar (Guideline Clearinghouse)<br>• Profesyonel Organizasyonlar (AAFP, La Leche League, NIH	• Kullanım kolaylığı (“özet” ve “NNTs halihazırda hesaplanmış”)<br>• Kullanılabilirlik (“Çevrim-içi olarak kolaylıkla kullanılabilir”)	• Söz konusu hastaya uygulanabilir bilgiler (örneğin; “özel bir hasta ile ilgili ayrıntılar üzerinden geçilebilir” veya “çalışmaların çoğu Avrupa kaynaklı”)<br>• Söz konusu spesifik müdahaleleri içerir<br>• Özgünlük (genel bakış veya odaklanmış) (“temel bilgi elde edilebilir” veya “daha fazla uzmanlaşmış”)<br>• Kaynağın kapsamlılığı (Kaynakta cevap bulma olasılığı) (örneğin “her şey bulunabilir” veya “faydalı kaynaklar içerir” veya “bu soruyu cevaplaması mümkün değil”)	“1. Derece kanıt” veya “hiçbir kaynak sağlanmamıştır”)<br>• Uzman yanlılığı (örneğin; “genellikle sadece birinin görüşü”)<br>• Sistematik yaklaşım<br>• Akran değerlendirmesi<br>• Doğrulama yeteneği<br>• Standart bakım (örneğin: “tıp camiası tarafından kabul edilmiş”)<br>• Geçerliği değerlendirmek için yeterli bilgi (örneğin: “sadece özet” veya “tam-metin mevcut değil”)<br>• Güncel/güncel olmayan (örneğin; “en son araştırmalar”)

	web sitesi)<br>• Kişiler (meslektaş, konsültan, uzman, kütüphaneci)			
Güçlü<br>(4 puan)	Üç tip kaynak sıralanmış	Kolaylıkla ilgili bir spesifik konu/açıklama içerir	İlgililik ile ilgili bir spesifik konu/açıklama içerir	Geçerlik ile ilgili bir spesifik konu/açıklama içerir
Kısıtlı<br>(2 puan)	İki çeşit kaynak belirtilmiş	Bir veya daha fazla kaynak kullanıma ilişkin kolaylıktan bahseder ancak açıklama yapmaz.<br>Örneğin: “uygun”, “kolay” ya da “zor”	Bir veya daha fazla kaynağın ilgililiğinden bahseder ancak açıklama yapmaz.<br>Örneğin: İlgili	Bir veya daha fazla kaynağın geçerliğinden bahseder ancak açıklama yapmaz.<br>Örneğin: “iyi”, “önemsiz”
Belirsiz<br>(0 puan)	Çeşitlilik yok. Sadece bir kaynak belirtilmiş ya da belirtilen tüm kaynaklar aynı tip.	Kolaylıktan bahsedilmemiş	İlgililikten bahsedilmemiş	Geçerlikten bahsedilmemiş

3. Yukarıdaki klinik senaryolardan bir tanesine odaklanın (emzirme ve oral kontraseptifler ya da yatak ıslatma alarmı). Sizce hangi tür bir çalışma (çalışma tasarımı-study design) bu soruyu en iyi şekilde yanıtlar? Neden?

	**Çalışma Tasarımı**	**Gerekçe**
Mükemmel (12 puan)	En iyi kaynakların isimlerini verir: Randomize Kontrollü çalışma veya Randomize Çalışma, Sistematik Derleme veya Randomize Kontrollü Çalışmaları içeren Meta-analiz, Randomize Çift Kör Klinik Çalışmalar	Randomizasyonun ve/veya körlemenin önemini anladığını gösteren mantıklı bir açıklama yapar. Randomizasyon ile karıştıcı faktörlerin azalması arasında ve/veya körleme ile gözlemci veya ölçüm yanlılığının azalması arasında açıkça bağlantı kurabilir. Örneğin: "Randomize kontrollü çalışma randomizasyon sayesinde sonucu etkileyebilecek yanlılıktan kaçınılmasını sağlayacaktır" veya "Yanlılığı azalttığı ve karıştırıcı faktörleri kontrol edebildiği için tedavi soruları için en uygun çalışma tipidir"
Güçlü (9 puan)	Yukarıda olduğu gibi en iyi kaynaklardan birisinin ismini veremez ama açıklama yapabilir<br><br>Örneğin: "iki grup karşılaştırılır, bir gruba tedavi alır, diğer gruba placebo verilir…"	Gerekçe mevcuttur, randomizasyon ve/veya körleme ile ilgili konulara değinir ancak daha az açıklıkta ifade edilmiştir.<br><br>Örneğin: "gruplar benzer olmalıdır" veya "karıştırıcı faktörleri ortadan kaldırmaya çalışır" veya "seçim yanlılığından kaçınır" veya "objektif olmak" veya "yanlılığı ortadan kaldırmak"
Kısıtlı (6 puan)	Daha az tercih edilen bir çalışma tasarımını açıklar ya da ismini verir.	Gerekçe mevcuttur, maliyet etkinliği, etik kaygılar, anımsama yanlılığı gibi randomizasyon ve körleme ile alakalı olmayan meşru konulara değinir. Randomizasyon ya da körlemeden bahsedebilir ancak açıklama yapmaz (randomize ve kör düzenlemelerde en iyidir)

	Örneğin: “Kohort çalışma” veya “prospektif klinik çalışma” “benzer çalışmaların meta-analizi” “longitudinal” veya “prospektif”	Örneğin: “doğum kontrol yerine placebo alacak kadınları çalışmaya dahil etmek imkansızdır” veya “ hasta dosyalarının incelenmesi çok fazla maaliyeti olmaksızın birçok veri sağlar”
Minimum (3 puan)	Tedavi sorusunu açıklamakta yetersiz olan bir çalışma tasarımını açıklar ya da ismini verir.<br><br>Örneğin: vaka kontrol, kesitsel çalışma, vaka sunumu, “retrospektif”<br>Ya da yetersiz ayrıntılar vererek bir çalışma tasarımını tanımlar:<br>Örneğin: Karşılaştırma çalışması	Gerekçe göstermeye çalışır fakat görüşleri çalışma tasarımı ve geçerliği tehdit faktörler arasındaki ilişkiyi anladığını göstermez ve non-spesifiktir. Randomizasyon veya körlemeden açıklama yapmadan bahsedebilir. (örn: randomize ve kör düzenlemeler en iyidir)<br><br>Örneğin: “kaliteyi sağlamak için” veya “olası çatışmaları azaltmak için” veya “karşılaştırmak için”
Belirsiz (0 puan)	Yukarıdakilerin hiçbiri mevcut değil	Yukarıdakilerin hiçbiri mevcut değil

4. Bu sorulardan birisi ile ilgili özgün bir araştırmayı Medline'da arayacak olsanız; nasıl bir tarama stratejisi izlerdiniz, açıklayınız. Hangi konular ve araştırma kategorilerini (alanları) tarayacağınızı belirtiniz. Neden bu yaklaşımı seçtiğinizi açıklayınız. Taramanızı sınırlamanız (limit) gerekirse bunu nasıl yaparsınız açıklayınız ve gerekçesini anlatınız.

	**Arama Terimleri**	**Etiketler**	**Sınırlayıcılar**
Mükemmel (8 puan)	Hasta (Patient), müdahale (intervention), karşılaştırma (comparison) ve sonucu (outcome) yansıtan 3 veya daha fazla terim.	Arama stratejisi ile ilgili açıklaması makalelerin veri tabanlarında birden fazla alana göre indekslendiğini anladığını gösterir.<br><br>Bir veya birden fazla alan/indeks /etiket ismi vererek tartışır ve tarama stratejisi için bu indekslerden bir veya daha fazlasını kullanarak makul bir gerekçe sağlar (MeSH, başlık kelimesi, yayın başlığı, anahtar kelime, yazar, dergi ismi)	Aramayı sınırlandırmakta kullanılan yaklaşımların birden fazlasını açıklar (örneğin; "insan sınırlandırması" veya "yetişkin" veya "İngilizce").<br><br>Spesifik bir yayın tipinin ismini verir veya PubMed'deki Klinik Sorgular ("Clinical Queries") veya Boolean operatörlerinin kullanımını veya arama kombinasyonlarını açıklar veya en iyi çalışma tasarımı ile ilgili bir terimi ekler (örn: randomize) veya alt başlık kullanımını önerir.<br><br>indeks ismi de verirse hem etiket için hem de sınırlama metodu için puan verin.

		Örneğin: anahtar kelime MeSH'e göre daha az spesifiktir.	NOT: Eğer öğrenci bir sınırlayıcıyı açıklarken (yabancı dilin İngilizce olarak işaretlenmesi)
Güçlü (6 puan)	PICO'dan iki terim	Bir ya daha fazla alan veya indeks kategorisinin ismini verir ancak arama stratejisini bu bilgiye dayalı olarak mantıklı bir şekilde savunamaz<br><br>Örneğin: "anahtar kerime taraması yapardım"	Aramayı sınırlamada kullanılan genel bir metodu açıklar
Kısıtlı (3 puan)	PICO'dan bir terim	Uygulanabilir değil	Uygulanabilir değil
Belirsiz (0 puan)	Mevcut değil	Makalelerin farklı alanlar ya da indekslerce etiketlendiği yönünde belirgin bir anlayış sergileyemez.	Listelenen aramayı sınırlamak için geçerli bir yöntem mevcut değil.

5. Bu sorularla ilgili özgün bir araştırma makalesi bulduğunuzda, bunun araştırdığınız konu ile ilgili olup olmadığını (relevant) saptamak için araştırmanın hangi özelliklerine dikkat edersiniz? Örnekler veriniz. (6 ve 7. sorularda çalışmanın geçerli bir çalışma olup olmadığını ve bulguların ne kadar önemli olduğu sorulacaktır. Bu soru için sizin pratik uygulamanızla gerçekten ilgili olup olmadığını nasıl belirleyeceğinizi düşününüz).

(5-7.sorular ilgililik, geçerlik ve etki büyüklüğü alt başlıklarına ayrılmış olan literatürün eleştirel değerlendirilmesi konusuna odaklanmaktadır. Bunlar eleştirel değerlendirme sürecinin rastgele seçilmiş alt başlıklarıdır. Dolayısıyla katılımcılar ilgililik ile alakalı konuları bu üç sorunun her hangi birisinde yanıtlayabilirler. Yönergede bulunan kriterleri uygularken bu üç soruya verilen yanıtları tek bir yanıt olarak değerlendiriniz.)

	**Soru**	**Öğrencilerin Açıklamaları**
Mükemmel<br>(12 Puan)	Çalışmada kullanılan bağımlı ve bağımsız değişkenler üzerinden örnekler verip, spesifik nedenleri açıklayarak ilgililik ile alakalı iyi açıklamalı ve özenli bir tartışma gerçekleştirir.<br>Şunlardan bahsedebilir:	Şunların ikisini de içerir:

	• Test ya da müdahalenin fizibilitesi<br>Örneğin: " bu test işe yarayabilir ancak benim uygulamalarımın makineyi satın almaya gücü yetmezse anlamı olmaz"<br>• Sonucun hasta ya da hastalık odaklı olması<br>Örneğin: Bir hafta ya da aylar sonra yatak ıslatılmayan gece sayısı belirlenmiş midir? ya da "sadece üretilen süt miktarı değil bebeğin büyümesi de takip edilmelidir"<br>• Araştırma sorusu ve işlemsel tanım arasındaki uyum<br>Örneğin; "ölçme yöntemlerinin bizim önemsediğimiz sonucu gerçekçi olarak yansıtıp yansıtmadığı"	• Çalışma denekleri ile hedef popülasyon arasındaki bağlantının önemini açık bir şekilde ifade eder.<br>• İlgili, hastalık ya da demografik özellikler ile ilgili en az bir örnek verir<br><br>Örneğin: "Hastalar yaş ve ırk bakımından benim hastalarımla benzer mi?" veya "hastane ya da kliniğin örneklemi benim hastalarımla benzer mi?" veya "hastalıkların şiddeti benim hastalarımınkiyle aynı düzeyde mi?" veya "seçim ya da uygun olmayan dahil etme kriterleri bu çalışmanın popülasyonunu yaş, ırk vs. bakımından benimkinden farklı kılmış mı?"
Güçlü<br>(9 Puan)	Çalışmada kullanılan bağımlı ve bağımsız değişkenlerin ilgili olması ile alakalı daha az düşünceli bir tartışma gerçekleştirir. Belli bir mantık içermeyen kavramlar ya da örnekleri ekleyebilir. Şunlardan bahsedebilir:<br><br>• Test ya da müdahalenin fizibilitesi.<br>Örneğin; "uygun mu?" veya "bunu kullanabilir miyim?"<br>• Sonucun hasta ya da hastalık odaklı yapıda olması.<br>Örneğin: "hasta odaklı sonuçlara bakmak" veya "sonuç benim hastam için önemli midir?"	Şunlardan bir tanesini içerir ancak ikisini birden değil:<br><br>• Çalışma denekleri ile hedef popülasyon arasındaki bağlantının önemini açık bir şekilde ifade eder.<br>• İlgili, hastalık ya da demografik özellikler ile ilgili en az bir örnek verir<br><br>Örneğin: "Hasta benim hastam gibi mi?" veya "popülasyonun eğitim seviyesi"

	• Araştırma sorusu ve işlemsel tanım arasındaki uyum.<br>Örneğin; çalışma için planladıkları şeyleri ölçmüşler midir?” veya “laktasyon performansını değerlendirmek için hangi yöntemleri kullanmışlardır?”	
Kısıtlı<br>(5 puan)	Yanıt çalışmanın eldeki soruya ne kadar hitap ettiğini değinir ancak neden önemli konusunda sunduğu tartışma yetersizdir.<br><br>Örneğin: “değişkenler nelerdir?”, “benim soru mu yanıtlıyor mu?”, “sonuç ölçütü”, “çalışmanın amacı”, “benim pratiğimi etkileyecek mi? ”bu çalışmada ne tür bir oral kontraseptif kullanılmıştır?”, “takip süresi ne kadardır?”	Yanıt çalışmaya katılan denekleri dikkate alır ancak denekler ile hedef popülasyon ya da örneklemin spesifik özellikleri arasında bağlantı kuran bir tartışma sunamaz.<br><br>Örneğin: “uygun örneklem midir?”, yanıt verme ya da katılım oranı neydi?”, “dışlama kriterleri nelerdi?” veya “seçim yanlılığı” veya “düzenek” veya “çalışmanın yapıldığı yer”
Belirsiz<br>(0 puan)	Araştırma sorusu ve değişkenler hakkında herhangi bir tartışma yok.	Araştırma katılımcıları hakkında herhangi bir tartışma yok.

6. Yaptığınız taramada sorularınızla ilgili özgün bir araştırma makalesi bulduğunuzda, araştırmanın hangi özellikleri bulgularının geçerli olduğunu düşünmenizi sağlar? Örnekler veriniz (İlgili olma konusunu zaten ele aldınız, 7. soruda ise bulguların önemini nasıl değerlendireceğiniz sorulacaktır. Bu soru için çalışmanın geçerliğine odaklanınız).

(5-7.sorular ilgililik, geçerlik ve etki büyüklüğü alt başlıklarına ayrılmış olan literatürün eleştirel değerlendirilmesi konusuna odaklanmaktadır. Bunlar eleştirel değerlendirme sürecinin rastgele seçilmiş alt başlıklarıdır. Dolayısıyla katılımcılar geçerlik ile alakalı konuları bu üç sorunun her hangi birisinde yanıtlayabilirler. Yönergede bulunan kriterleri uygularken bu üç soruya verilen yanıtları tek bir yanıt olarak değerlendiriniz.)

	**İç Geçerlik**
Mükemmel<br>(24 puan)	İç geçerlik için önemli olan en az 5 konuyu sıralar ya da açıklar. Örneğin:<br>• Çalışma tasarımının uygunluğu<br>• Körlemenin yeterliliği<br>• Randomizasyonun gizlenmesi<br>• Grup atamalarının randomize olması<br>• Geçersiz ya da yanlı ölçümler (“kendi protokolü izlendi mi?)<br>• Karşılaştırma veya kontrol grubunun önemi<br>• Tedavi amacına yönelik analiz<br>• Uygun değişkenlerin göz önünde bulundurulması (“ilgili diğer faktörler göz önünde bulundurulmuş mudur?”)

	• Varılan sonuç kanıtla uyumlu mudur? (“bulgular anlam ifade eder mi?”)<br>• Çalışmaya dahil edilen tüm katılımcıların takibinin önemi<br>• Uygun istatistiksel analiz<br>• Örneklem büyüklüğü / Güç<br>• Sponsorluk<br>• Çalışmanın yapıldığı zaman<br>• Diğer çalışmalar tarafından onaylanması
Güçlü<br>(18 puan)	Yukarıdaki konulardan 3-4 tanesini tanımlar
Kısıtlı<br>(10 puan)	Yukarıdaki konulardan 2 tanesini tanımlar
Çok az<br>(5 puan)	İç geçerlikten bahseder ya da yukarıdaki örneklerde yer alan spesifik kavramlardan birisini belirtir.
Belirsiz<br>(0 puan)	Yukarıda belirtilenlerden hiçbirisi mevcut değildir

7. Sorularınızla ilgili özgün bir araştırma makalesi bulduğunuzda, bulguların hangi özellikleri onların kuvvet (etki büyüklüğü) ve istatistiksel anlamlılığını belirlemenizi sağlar? Örnekler veriniz (İlgili olma ve geçerlilik konusunu zaten ele aldınız. Bu soru için çalışmada bildirilen bir etkinin büyüklüğünü ve anlamını nasıl belirleyeceğinize odaklanınız)

(5-7.sorular ilgili olma, geçerlik ve etki büyüklüğünün şiddeti alt başlıklarına ayrılmış olan literatürün eleştirel değerlendirilmesi konusuna odaklanmaktadır. Bu başlıklar kritik değerlendirme sürecinin rastgele seçilmiş alt başlıkları olabilir. Dolayısıyla katılımcılar kuvvet ve anlamlılık ile ilgili konuları bu üç sorunun her hangi birisinde yanıtlayabilirler. Yönergede bulunan kriterleri uygularken bu üç soruya verilen yanıtları tek bir yanıt olarak değerlendiriniz)

	**Etki Büyüklüğü**	**İstatistiksel Anlamlılık**
Mükemmel<br>(12 puan)	Yanıtta açık olarak şunların <u>ikisinin de</u> tartışılıyor olması gerekir:<br>• Klinik anlamlılık (“klinik anlamlılık nedir? Veya “ne kadar büyük fark bulunmuştur”)<br>• Etki büyüklüğünün ölçümü ile ilgili örnek/örnekler (örneğin: spesifite, sensivite, testin olabilirlik oranı, tedavi için gerekli sayı, rölatif risk, mutlak risk azalması, sürekli değişkenler için ortalama fark, pozitif veya negatif öngörü değeri)	İstatistiksel anlamlılık konusuyla ilgili önemli kavramlara ait en az iki spesifik örnek vererek mantıklı ve dikkatli açıklama yapar.<br>• p-değeri<br>• Güven aralığı<br>• Güç<br>• Tahminlerin kesinliği<br>• Tip 1 ve Tip 2 hata

Güçlü<br>(9 puan)	Yanıt şunlardan ikisini değil bir tanesini tartışmaktadır<br>• Klinik anlamlılık ("klinik anlamlılık nedir? Veya "ne kadar büyük fark bulunmuştur")<br>Etki büyüklüğünün ölçümü ile ilgili örnek/örnekler (örneğin: spesifite, sensivite, testin olabilirlik oranı, tedavi için gerekli sayı, rölatif risk, mutlak risk azalması, sürekli değişkenler için ortalam fark, pozitif veya negatif öngörü değeri)	Yukarıdaki kavramlardan birisini yetersiz olarak açıklar ya da hiç açıklamaz (Örneğin: "p-değeri veya güven aralığı") veya<br>Sadece bir kavramı sayar ve tartışır (Örneğin: "p-değerinin 0.05 den küçük olması")
Sınırlı<br>(5 puan)	Yanıt sadece klinik anlamlılık ya da etki büyüklüğünü göz önünde bulundurur.<br>(Örneğin "bu önemli mi?", " benim pratiğimi etkileyecek mi?"	
Belirsiz<br>(0 puan)	Yukarıdakilerden hiçbirisi mevcut değildir	Yukarıdakilerden hiçbirisi mevcut değildir

8. Pulmoner emboli tanısı koymada arter kan gazı değerlerinin tanısal duyarlılığı ile ilgili yeni yapılan bir çalışmaya, pulmoner emboli kuşkusu olan 212 hasta dahil edilmiştir, daha sonra bu hastaların 49'unda pulmoner emboli olduğu saptanmıştır. Pulmoner embolisi olanların 41 inde anormal alveol-arter oksijen gradiyenti ((A-a) DO_2) olduğu görülmüştür. Pulmoner embolisi olmadığı saptanan 163 hastanın 118 inde anormal (A-a) DO_2 olduğu belirlenmiştir.

Bu sonuçlara dayanarak pulmoner emboli için aşağıdaki hesaplamaları yapınız (Her biri 4 puan).

- Bu sonuçlara dayanarak, pulmoner emboli için (A-a) DO_2 sensivitesi: 0,837 VEYA 41/49_
- Bu sonuçlara dayanarak, pulmoner emboli için (A-a) DO_2 spesifitesi: 0,276 VEYA 45/163 _
- Bu sonuçlara dayanarak, pulmoner emboli için (A-a) DO_2 pozitif öngörü değeri: 0,258_ VEYA 41/159 VEYA 41/(41+118)
- Bu sonuçlara dayanarak, pulmoner emboli için (A-a) DO_2 negatif öngörü değeri: 0,849_ VEYA 45/53 VEYA 45/(8+45)
- Bu sonuçlara dayanarak, pulmoner emboli için anormal (A-a) DO_2 pozitif olabilirlik oranı: 1,156 VEYA 0,84/(1-0,28)

9. Yakın zamanda yapılan randomize bir çalışmada koroner arter hastalığı olan diyabet hastaları 5 yıl takip edilmiştir. Pravastatin ile tedavi edilen hastaların % 29'u tekrar eden koroner hadiseden yakınırken plasebo grubundakilerin %37'sinin tekrar eden koroner hadiseden yakındığı saptanmıştır.

   Tekrarlayan hadise için aşağıdaki değerleri belirleyiniz (Her biri 4 puan)

   - Tekrarlayan hadise için mutlak risk azalması 8% VEYA 0,37-0,29
   - Tekrarlayan hadise için rölatif risk azalması 22% VEYA (0,37-0,29)/.37 VEYA ,08/0,37 VEYA 1-(0,29/0,37)
   - Bir tekrarlayan hadiseyi önlemek için tedavi edilmesi gereken hasta sayısı (NNT) 12,5 VEYA 1/0,08 VEYA 1/(0,37-0,29)

10. Yakın zamanda yapılan HERS çalışmasında östrojen desteği alan kadınlar ile plasebo alan kadınlar karşılaştırılmıştır. Sonuçlar, östrojen kullanan kadınlarda venöz tromboembolik hadise için rölatif riskin 2.89 olduğunu göstermiştir. Bu sonuç, östrojen tedavisinin koroner arter hastalıkları için risk oluşturduğunu düşündürmektedir. Bu iki tedavi grubu arasındaki farklılığın istatistiksel olarak anlamlı olup olmadığını belirlemek için güven aralığına bakıyoruz. Venöz trombolembolik hadise oranının gerçekten (istatistiksel olarak) bu iki tedavi grubu için farklı olduğu çıkarımını destekleyecek güven aralığına bir örnek veriniz.

    2,89' u kapsayan ve 1'i içermeyen herhangi bir değer

11. Size göre tanıya (diagnosis) yönelik bir araştırma için en iyi çalışma tasarımı nedir?

    Kesitsel çalışma VEYA testin altın standard ile karşılaştırılması

12. Size göre prognoza (prognosis) yönelik bir araştırma için en iyi çalışma tasarımı nedir?

    Kohort çalışmalar VEYA prospektif VEYA longitudinal
